# Characterization of an N-terminal Na_v_1.5 channel variant – a potential risk factor for arrhythmias and sudden death?

**DOI:** 10.1186/s12881-020-01170-3

**Published:** 2020-11-19

**Authors:** Stefanie Scheiper-Welling, Paolo Zuccolini, Oliver Rauh, Britt-Maria Beckmann, Christof Geisen, Anna Moroni, Gerhard Thiel, Silke Kauferstein

**Affiliations:** 1 Institute of Legal Medicine, Goethe University of Frankfurt, Kennedyallee 104, 60596 Frankfurt am Main, Germany; 2 Department of Biology, Membrane Biophysics, Technische Universität Darmstadt, Schnittspahnstrasse 3, 64287 Darmstadt, Germany; 3 1 Institute of Legal Medicine, Goethe University of Frankfurt, Kennedyallee104, 60596 Frankfurt am Main, Germany; 4 German Red Cross Blood Center, Institute of Transfusion Medicine and Immunohaematology, University Hospital Frankfurt, Frankfurt, Germany; 5 Department of Biosciences and CNR IBF-Mi, University of Milano, Via Celoria 26, 20133 Milan, Italy

**Keywords:** Arrhythmia syndromes, Sudden death, Cardiac arrest, *SCN5A*, Functional characterization

## Abstract

**Background:**

Alterations in the *SCN5A* gene encoding the cardiac sodium channel Na_v_1.5 have been linked to a number of arrhythmia syndromes and diseases including long-QT syndrome (LQTS), Brugada syndrome (BrS) and dilative cardiomyopathy (DCM), which may predispose to fatal arrhythmias and sudden death. We identified the heterozygous variant c.316A > G, p.(Ser106Gly) in a 35-year-old patient with survived cardiac arrest. In the present study, we aimed to investigate the functional impact of the variant to clarify the medical relevance.

**Methods:**

Mutant as well as wild type GFP tagged Na_v_1.5 channels were expressed in HEK293 cells. We performed functional characterization experiments using patch-clamp technique.

**Results:**

Electrophysiological measurements indicated, that the detected missense variant alters Nav1.5 channel functionality leading to a gain-of-function effect. Cells expressing S106G channels show an increase in Na_v_1.5 current over the entire voltage window.

**Conclusion:**

The results support the assumption that the detected sequence aberration alters Na_v_1.5 channel function and may predispose to cardiac arrhythmias and sudden cardiac death.

**Supplementary Information:**

The online version contains supplementary material available at 10.1186/s12881-020-01170-3.

## Background

Cardiac sodium channels are responsible for the inward depolarizing current (I_Na_) in the initial phase of the cardiac action potential. The cardiac expressed gene *SCN5A* encodes the pore-forming ion conducting α-subunit of the voltage-gated sodium channel Na_v_1.5, which is highly conserved among species [1–4]. The Na^+^ current resulting from Na_v_1.5 is fundamental in initiation and propagation of action potentials and is therefore responsible for cardiac excitability and conduction velocity [2]. In cardiomyocytes, Na_v_1.5 is preferentially localized at intercalated discs and at the T-tubular system [1].

The sodium channel Na_v_1.5 consists of four homologous domains (DI, DII, DIII and DIV), each consisting of six membrane spanning segments (S1 - S6). The positively charged S4 segments act as voltage sensor and are responsible for the voltage-dependent ion channel activation. The pore of the sodium channel is formed by the extracellular loops (P-loops) between S5 and S6 segments. The N- as well as the C- terminal region of the α-subunit are located in the cytoplasm [3–7].

Genetic aberrations in *SCN5A* have been associated with different types of arrhythmias and diseases including Brugada syndrome (BrS), long-QT syndrome (LQTS), sick-sinus-syndrome (SSS), atrial fibrillation (AF), dilated cardiomyopathy (DCM) and non-cardiac diseases as for example epilepsy. However, abnormalities in the sodium channel can also lead to overlapping phenotypes, so called overlap syndromes [3, 6, 8–10]. Loss-of-function mutations have been associated with Brugada syndrome, whereas gain-of-function variants are known to cause long QT syndrome type 3. Next to those electrical diseases, loss- as well as gain-of-function variants were described in cases of the structural heart disease dilated cardiomyopathy. However, the precise mechanism leading to the DCM phenotype remains unclear [3, 10, 11]. The pathogenesis and clinical spectrum of *SCN5A* variants is quite complex. Whereas one patient is showing a severe phenotype, another patient carrying the same genetic variant exhibits no clinical conspicuities. Phenotypes may even vary within one family. In the worst case, sudden cardiac death (SCD) is the first sign of a hereditary disease. The variable disease severity, which is most likely influenced by environmental and genetic factors, remains incompletely understood yet [3, 10, 12].

In the present study, we identified the variant of unknown significance c.316A > G, p.(Ser106Gly) in the N-terminal region of the cardiac sodium channel Na_v_1.5 in a patient with survived cardiac arrest. Variants in the N-terminus of Na_v_1.5 have been identified in patients with BrS and LQTS. However, the role of the N-terminal region on trafficking, localization and regulation is not fully understood until now [13, 14]. In order to better assess the relevance of the detected variant, we performed functional studies in HEK293 cells.

## Methods

### Ethics statement

The study was conducted in accordance with the “Declaration of Helsinki”. The patient signed informed consent with approval for publication.

### Genetic analysis

DNA was extracted from blood using salting out procedure. Genetic screening was carried out by means of Next-generation sequencing (NGS). Library preparation was performed using Nextera Flex Technology combined with the TruSight Cardio Panel (Illumina) consisting of 174 genes with known cardiac associations (supplementary information). Subsequent sequencing was carried out on a MiniSeq System (Illumina). The resulting reads were aligned to the human reference genome GRCh37/hg19. Variant calling and further evaluation was performed using the Software GensearchNGS (Phenosystems). Variants were filtered according to a pre-established prioritization protocol as described previously [15]. Evaluation was mainly based on presumed functional impact on the protein and allele frequency. Genetic aberrations expected to disrupt protein function and with minor allele frequency below 0.2% in public available databases (The Genome Aggregation and Exome Sequencing Project databases) were further evaluated. Detected sequence variants were assessed using common databases (as e.g. public population databases, Human Gene Mutation Database) and applying in silico prediction tools (PolyPhen-2 [16], MutationTaster [17], SIFT [18], CADD [19]). The identified variant was confirmed by polymerase chain reaction (PCR) and standard direct sequencing by means of BigDye Terminator v1.1 chemistry and 3130*xl* Genetic Analyzer (Applied Biosystems). The resulting data were evaluated using SeqScape Software.

### Mutagenesis

For site-directed mutagenesis, the pcDNA3.1-EGFP vector comprising the wild cDNA sequence of *SCN5A* (hH1a isoform) was used (kindly provided by N. Neyroud, PhD, Inserm-Sorbonne Université Paris, France). The required sequence variant was induced using the QuikChange II XL Site-Directed Mutagenesis Kit (Agilent Technologies). The resulting constructs were verified by direct sequencing.

### Electrophysiological measurements

For the functional characterization of the variant p.S106G, the mutant as well as wild type Na_v_1.5 channels were expressed in human embryonic kidney (HEK) cells, cell line 293 as described previously [20]. To mimic the heterozygous state of the patient, wildtype and mutant channels were also co-transfected in a 1/1 ratio (WT/MT). HEK293 cells were cultivated at 37 °C, 5% CO2 and passaged by transferring them to 35 mm plates. Transfection was performed by means of TurboFect transfection reagent (Thermo Fisher Scientific, Waltham, USA) and 3 μg of Na_v_1.5 constructs in the pcDNA3.1-EGFP vector. On the next day, cells were detached using accutase (PAA, GE Health Freiburg, Germany). Four hundred microliters were transferred to a new plate comprising 2 ml medium. Electrophysiological measurements were performed on single cells approximately 36 h following transfection using conventional patch clamp technique. Current measurements were carried out at room temperature in whole-cell configuration. Measurements and data acquisition were performed with an EPC-9 Patch Clamp amplifier (HEKA, Lambrecht, Germany) and the Patch Master (HEKA) software. Data were low pass filtered at 10 kHz and sampled at 20 kHz. Currents were measured in a bath solution composed of 135 mM NaCl, 4 mM KCl, 1 mM CaCl_2_, 2 mM MgCl_2_, 20 mM Glucose and 10 mM HEPES (pH 7.4, adjusted with NaOH). The pipette solution consisted of 140 mM CsCl, 5 mM NaCl, 4 mM Mg-ATP, 2 mM MgCl_2_, 5 mM EGTA, 10 mM HEPES (pH 7.4, adjusted with CsOH). In order to measure the peak current amplitudes and determine current/voltage (I/V) relationships, the cells were clamped from a holding potential of − 140 mV (100 ms) to test voltages ranging from − 100 mV to + 50 mV in 5 mV steps. To analyze the steady-state inactivation, cells were clamped from a holding potential of − 100 mV (100 ms) to test voltages ranging from − 140 mV to − 20 mV by 10 mV increments (500 ms), followed by a 50 ms test pulse to + 20 mV. For data analysis, we utilized Igor Pro 6.03 software (WaveMetrics, Lake Oswego, OR).

For noise analysis, Na_v_1.5 WT and S106G channels were activated 200 times by a depolarizing voltage step from a holding potential of − 140 mV to − 20 mV or − 40 mV. Current traces were imported into MATLAB to calculate the mean current <I > (t), the current differences between successive traces ΔI_n_(t), the mean current differences <ΔI>(t), and the variance of the current differences σ^2^(t). <I > (t), ΔI_n_(t), <ΔI>(t), and σ^2^(t) were calculated with the following equations [21]:

1 $$ \left\langle \mathrm{I}\right\rangle \left(\mathrm{t}\right)=\frac{1}{\mathrm{M}}{\sum}_{\mathrm{n}=1}^{\mathrm{M}}{\mathrm{I}}_{\mathrm{n}}\left(\mathrm{t}\right) $$

2 $$ \Delta  {I}_n(t)=\frac{I_n(t)-{I}_{n+1}(t)}{2} $$

3 $$ \left\langle \Delta  I\right\rangle (t)=\frac{1}{\mathrm{M}-1}{\sum}_{\mathrm{n}=1}^{\mathrm{M}}\Delta  {I}_n(t) $$

4 $$ {\sigma}^2(t)=\frac{2}{\mathrm{M}-1}{\sum}_{\mathrm{n}=1}^{\mathrm{M}}{\left(\Delta  {I}_n(t)-\left\langle \Delta  I\right\rangle (t)\right)}^2 $$

where n is trace number and M the total number of traces collected. σ^2^ values were plotted against I_mean_ and fitted with the following parabolic equation:

5 $$ {\sigma}^2\left(\left\langle I\right\rangle \right)=i\bullet \left\langle I\right\rangle -\frac{{\left\langle I\right\rangle}^2}{N}+{\sigma_b}^2 $$

where i is the single channel amplitude, N the total number of channels, and σ_b_^2^ the background noise. Data were fitted with the constrain that σ_b_^2^ is ≥0.

### Data analysis

Results are reported as mean ± standard deviation (SD) of n experiments. An unpaired student’s t-test was applied to determine the statistical significance of the results obtained.

## Results

### Clinical background and genetic analysis

**Fig. 1:**
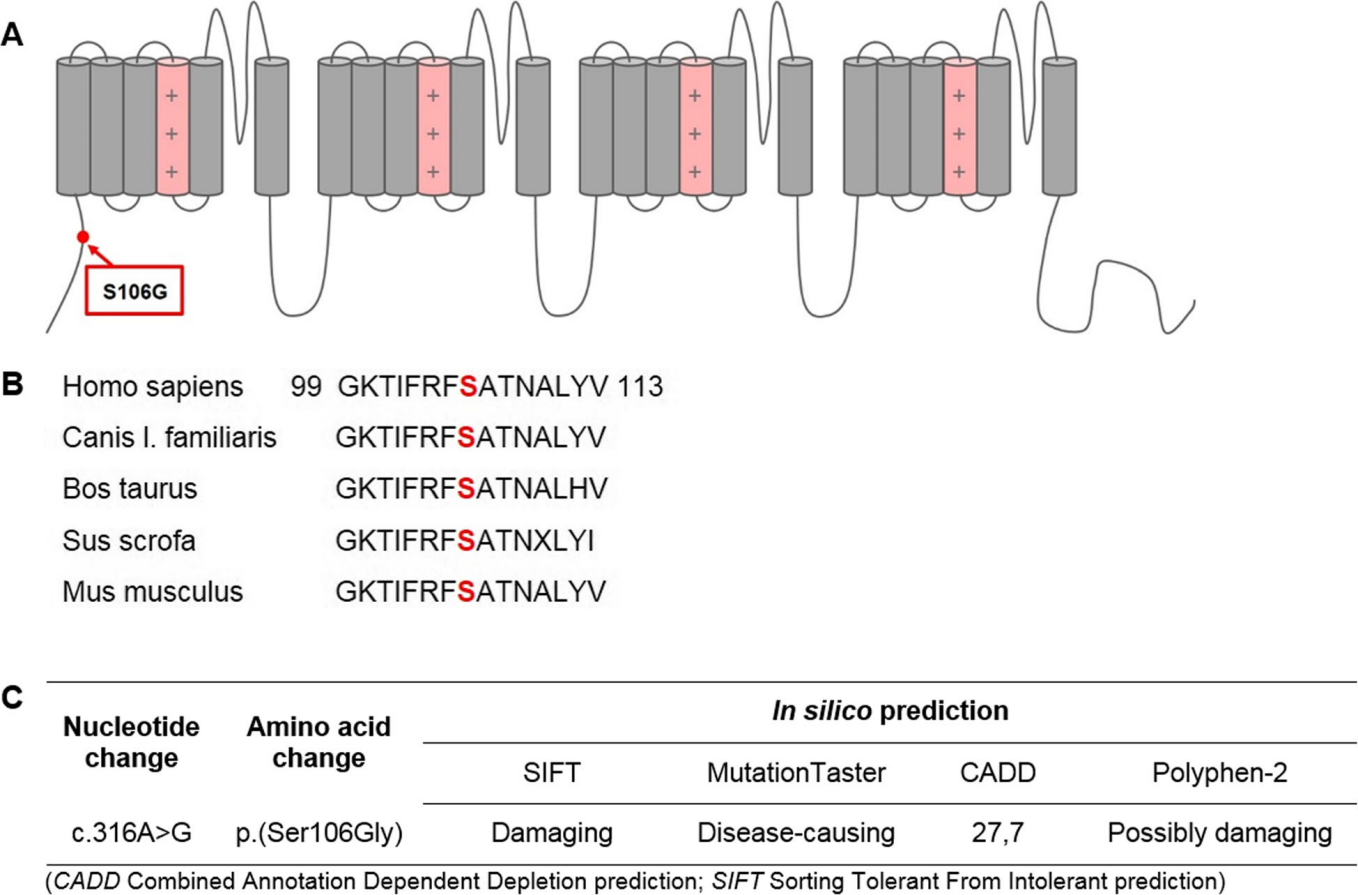
Detection of the *SCN5A* variant p.(Ser106Gly) in a patient with aborted cardiac arrest. **a** Schematic representation of the Na_v_1.5 channel and localization of the detected N-terminal variant. **b** Alignment of a partial N-terminal sequence among mammals. The affected amino acid is indicated in red. **c** Assessment of the genetic variant by in silico prediction tools

**Fig. 2:**
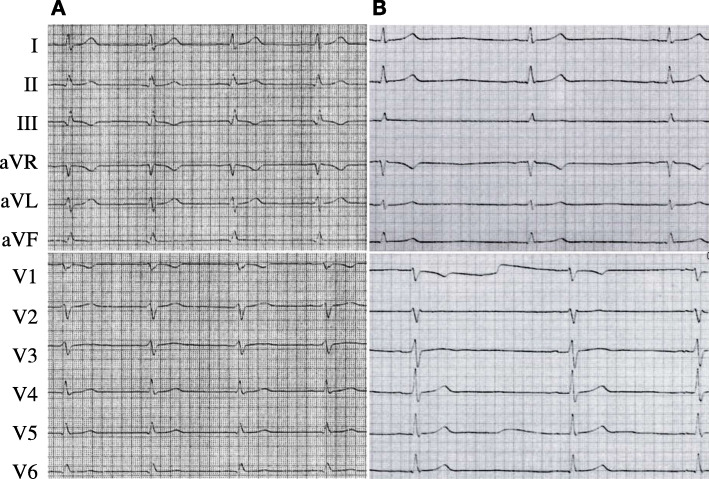
ECG findings of the patient. **a** ECG taken 1 year previous to the cardiac event is showing sinus rhythm with a heart rate of 66/min, flat P waves, PR interval 140 ms, QRS 100 ms, T inversion in lead III and QTc of 389 ms. **b** ECG from approximately 3 months after cardiac event under beta-blocker therapy is showing sinus bradycardia with a heart rate of 38/min, flat P waves, PR interval 190 ms, QRS 110 ms and QTc of 365 ms

Next-generation sequencing using a defined cardio panel revealed the heterozygous *SCN5A* variant c.316A > G, p.(Ser106Gly), rs1331765859, in a patient with survived cardiac arrest. This variant is localized in the cytoplasmic N-terminal region of the Na_v_1.5 channel (Fig. 1a). No further potentially informative rare variant (MAF ≤ 0.2%) was detected. The male patient experienced sudden cardiac arrest at the age of 35 and was finally resuscitated after 53 defibrillations. Cardiological examination revealed no distinct clinical findings and echocardiographic findings most likely pointed towards an incipient dilative cardiomyopathy as the left ventricular ejection fraction was initially slightly reduced (LV-EF 50–55%), but normalized quickly. Hence, this assumption could not be confirmed during follow-up investigations including cardiac magnetic resonance imaging (MRI). ECG tracings did not show specific findings and a provocative test with intravenous administration of the sodium channel blocker ajmaline in order to unmask a concealed Brugada syndrome was performed without displaying a diagnostic Brugada type I-ECG pattern (Fig. 2). So the cause for the cardiac arrest of the patient remained unclear and a arrhythmogenic cardiomyopathy could not be excluded. The patient stated that his father received the diagnosis of a cardiomyopathy, but unfortunately, he was not willing to provide more detailed clinical data and refused genetic analyses. The patient is also father of a toddler, but due to agitation, cardiological examination (ECG) was not possible until now. In order to prevent anxiety no genetic analyses will be performed prior to cardiological assesment due to missing specific cardiological consequences.

The amino acid serine (p.Ser106) in the intracellular N-terminal region of the Na_v_1.5 channel is highly conserved in mammals (Fig. 1b). The sequence variant is not listed in the common databases Exome Variant Server, ExAC (Exome Aggregation Consortium) or GnomAD (The Genome Aggregation Database) indicating that this is a very rare variant. It is only listed in the TOPmed database (Trans-Omics for Precision Medicine). The frequency stated in this database is 0.001% (in 125,568 alleles). Quite recently, the variant has been described in a sudden unexpected death of a 31 year old woman [22]. Aberrations in adjacent regions (p.Arg104Gln; p.Arg104Trp; Asn109Lys) have been associated with Brugada Syndrome [13, 14, 23].

In order to further assess the possible impact of the variant, several in silico predictions were applied. All tools were pointing towards a possible causative impact on the protein (Fig. 1c). Due to missing functional as well as co-segregation studies, this variant was classified as variant of unknown significance (VUS) according to the American College of Medical Genetics (ACMG) standards [24].

### Electrophysiological measurements in HEK293 cells

**Fig. 3:**
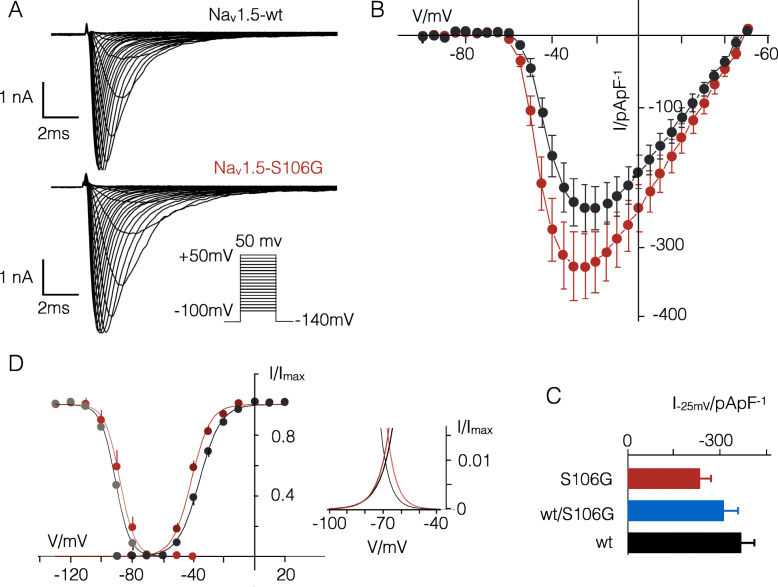
Na_v_1.5 S106G results in gain of function. **a** Typical Na^+^ currents in HEK293 cells transiently expressing Na_V_1.5 WT (top) or Na_V_1.5-S106G mutant (down). Currents were elicited by clamping cells from holding voltage (− 140 mV, 100 mS) to test voltages (between + 50 mV and − 100 mV, 100 ms) and back to post clamp voltage (− 140 mV, 100 ms). **b** Mean current density/voltage relationships (±SE) from cells transfected with WT (black, *n* = 9) or S106G mutant (red, *n* = 8). **c** Peak currents at − 25 mV from (**b**) for WT (black) mutant (red) and data from cells co-transfected with WT and S106G mutant (blue, *n* = 12). **d** Boltzmann fits of voltage-dependent inactivation (left) and activation (right) for Na_v_1.5 WT (black/grey) and Na_v_S106G mutant (red/brown). The mutation causes a positive shift in the V_1/2_ value for inactivation (− 88.2 ± 0.8 mV versus 91.5 ± 1 mV) and a negative shift of the V1/2 value for activation (− 45.3 ± 0.9 mV versus − 39.9 ± 0.8 mV). The *P* value for a difference between V_1/2_ values of the WT and mutant < 0.05. Inset: estimated window currents for WT and mutant from (**b**). The colors of the line correspond to those in (**c**)

In order to examine the functional relevance of the Na_V_1.5 variant p.Ser106Gly, wild type (WT) or mutant (MT) Na_v_1.5 channels were expressed with an n-terminal GFP-tag in HEK293 cells. The representative current traces in Fig. 3a show that whole-cell patch clamp recordings of GFP positive cells exhibit in all cases large, Na_V_-type, inward currents. When expressed at equivalent levels, the Na_v_1.5-S106G generated current was higher relative to that of the WT channel (Fig. 3b). At a reference voltage of − 25 mV the current density of the mutant exceeded the control value by a factor of 1.6 (*P* < 0.05). The density of the Na^+^ current exceeded that of the control by a factor of 1.3 and fell in between control and mutant when both were transfected together (Fig. 3c). Because of a high variability of the measured current densities this difference is not significant.

The S106G mutation also exhibited small effects on the voltage dependency of the channel. The V_0.5_ value for voltage-dependent inactivation is shifted by 3.3 mV positive relative to the WT channel (− 45.3 mV (*N* = 8) versus − 39.9 mV (*N* = 9) respectively). The respective value for voltage dependence activation is in the mutant shifted negative by 5.4 mV (− 91.5 vs. -88.2; *P* < 0.05 Fig. 3d). Combined with the shift in the inactivation curve this predicts a slight increase and right shift of the window current, which is available during excitation (Fig. 3d). This combination of elevated current density and shift of the voltage dependency of the channel is expected to elevate the Na^+^ load of cardiomyocytes. We also determined the same parameters for activation and inactivation in conditions in which WT and MT channel were expressed together (Tab. 1). The results show that the curves for activation and inactivation tend towards those of the mutant. They are significantly different (*P* < 0.05) from the reference values of the WT channel.

**Fig. 4:**
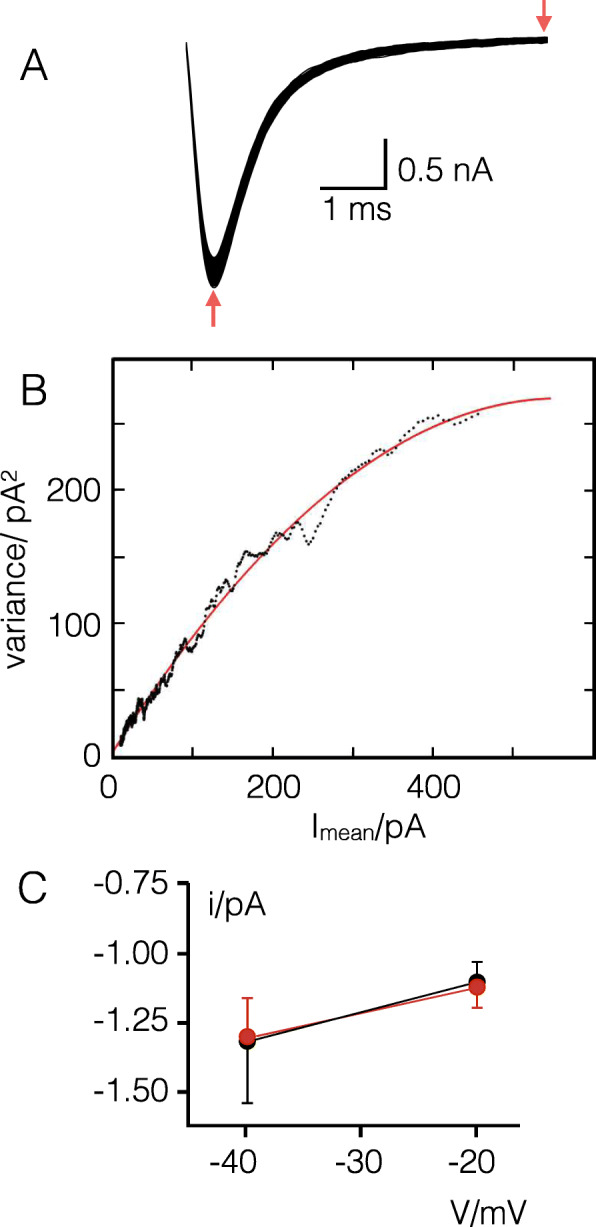
S106G mutation does not affect unitary channel conductance. **a** Exemplary recordings of a HEK293 cell expressing the Na_v_1.5 WT channel for noise analysis. Channels were activated 200 times by stepping membrane from holding voltage (1–40 mV, 100 ms) to test voltage of − 20 mV (100 ms). Red arrows delimit the part of the traces used for the analysis. **b** Example of noise analysis from one cell expressing Na_v_1.5 WT channel. The variance between pulses is plotted as a function of the mean current value. The data were fitted with parabolic function (Eq. 5) yielding the unitary channel conductance from the initial slope of the fit. **c** Average i values (mean ± SE) for WT and S106G channels at two different voltages: − 20 mV (WT *n* = 3, black; S106G *n* = 4, red) and − 40 mV (WT *n* = 4, black; S106G *n* = 4, red)

The increase in current density of the mutant relative to the WT over the entire voltage spectrum could originate from an increase in unitary conductance or an increase in the number of channels. To examine whether the mutation affects the unitary conductance we estimated the single channel amplitude by non-stationary noise analysis [25] in cells expressing the WT channel, the S106G mutant or both. For the noise analysis we considered the dynamics of channel inactivation from the peak current to the baseline (Fig. 4a). To eliminate unwanted changes in the leak conductance we considered for each isochrone the variance in currents between couples of subsequent voltage steps rather than the variance of the absolute values (see materials and methods). For each cell we plotted the variance values as a function of the mean current. The bell-shaped distribution could be fitted with the parabolic Eq. 5 (an example is reported in Fig. 4b). The first derivative of the function at the current minimum is a good estimate of the single channel amplitude *i* [25].

For the WT and S106G construct we performed a noise analysis for clamp steps from holding voltage of − 140 mV to − 20 mV and − 40 mV. The data in Fig. 4 show that the estimated single channel amplitudes (*i*) and the respective unitary conductance values are all very similar. The results of these experiments imply that the mutation has no impact on the unitary conductance of Na_v_1.5. This result is not surprising considering that the mutation is far from the pore of the channel.

## Discussion

Aberrations in the *SCN5A* gene have been described to cause disease by altered Na_v_1.5 structure, function or expression level. Various types of arrhythmias and disorders have been associated in the last decades with alterations in this gene. In our study, we describe a rare *SCN5A* variant, which might represent a potential risk factor for fatal arrhythmias and sudden cardiac death. In the present case, we detected the N-terminal missense variant p.(Ser106Gly) in a patient with aborted cardiac arrest. During follow-up investigations, the patient did not exhibit distinct clinical conspicuities. Quite recently, this variant has been described in a case of sudden unexpected death in a 31 year old woman [22]. It was known that she suffered from syncope, but showed no ECG abnormalities or diagnostic echocardiographic findings prior to death.

The results of the present experiments support the view that the mutation has an impact on channel function and that this could be causally related to life threatening arrhythmias. The electrophysiological characterization shows that the amino acid replacement in the N-terminus of the channel has a gain of functional impact on the channel. The most obvious functional gain is an increase in steady state current. This is functionally also relevant in a heterozygotic patient because mixed expression of WT and MT still elevates the steady state current relative to the WT channel. The mutation has also small impacts on the voltage dependent parameters with the effect that the window between activation and inactivation is slightly increased and right shifted. In analogy to the effect of other mutants of Na_v_1.5 [5] the combination of these parameters implies that the Ser106Gly mutation could increase in this manner the influx of Na^+^ and elevate the Na^+^ lode of cardiomyocytes. The electrical data do not yet explain the disease phenotypes of the patient carrying the mutant. But they underscore that the mutant causes some aberrant functions, which could be causally linked to heart arrhythmias.

Previous studies have already reported a vast number of mutations in the Nav1.5 channel [5, 26, 27]. The majority of these mutations are localized in the transmembrane domains of the protein, where they affect mostly gating features of the channel. In a second group the mutations are found, like in the present case, in cytosolic domains and associated with signaling or interactions with scaffolding proteins [28]. In the context of recent publications the phenotype of the present mutant may however be better explained by a third category namely mutations in which an interaction of cytosolic domains is important for channel trafficking [13, 14]. A contribution of phosphorylation/dephosphorylation of the critical Ser106 to this mechanism seems not relevant. A bioinformatics analysis suggests that several surrounding residues namely Thr101, Thr108 and Ser115 are potential targets of kinases while Ser106 is not [29].

It is interesting to note that the critical S106G mutation is located between two cationic amino acids R104 and R121 in the Nav1.5 channel [13]. Mutations of these two amino acids exhibit the exact opposite effect of the S106G mutation: they decrease the current density and shift the activation curve positive. As an explanation for the decrease in channel density in the R104W and R121W mutants a quality control mechanism was proposed [13]. According to this mechanism the N-termini of Nav1.5-subunits interact in the ER independent on the critical amino acids. When a mutation in one of the binding partners is mis-folded as a result of a mutation, the protein complex is retained in the secretory pathway and degraded. This quality control in the secretory pathway could reduce trafficking of channel proteins to the plasma membrane. It is tempting to speculate that the S106G mutation has the opposite effect in that it accelerates the quality control mechanism and augments in this manner trafficking of the channels to the plasma membrane. Such an increase in the number of channels would be in agreement with the experimental data, which show an increase in Na_v_1.5 current over the entire voltage window.

The underlying mechanisms of *SCN5A* mutations leading to gain-of-function effects are mainly due to abnormalities in channel kinetics. Various studies describe, that gain-of-function variants can cause many individual phenotypes, ranging from LQTS, DCM as well as unclear clinical phenotypes and in the worst case sudden death. Frequently, multiple phenotypes, so called overlap syndromes, are described for one and the same *SCN5A* mutation, which could also be explained by different electrophysiological properties of the channel in different areas of the heart. Furthermore, it is generally assumed, that there are other unknown mechanisms that are for instance related to age, co-morbidities or environmental influences that contribute to genotype-phenotype interactions. Identification of other modulators and genetic factors may help to understand how alterations in *SCN5A* can lead to so many diverse clinical phenotypes [3, 12, 26].

Based on the electrophysiological data, the severe cardiac event of the patient, in whom the variant was detected and last, the fact that the variant was identified in a further sudden death case of a young woman, who did not show any ECG abnormalities prior to death, conspicuously [22], the n-terminal variant has to be considered as a risk factor for fatal arrhythmias and sudden death.

## Conclusions

In the present study, we describe an electrophysiological characterization of the very rare sequence variant Ser106Gly in the *SCN5A* gene leading to a gain-of-function impact on the sodium channel Na_v_1.5. As described in previous studies, our data underline the assumption that the cytosolic n-terminal domain is probably important for channel trafficking. The results indicate, that the exchange of Ser for Gly is not recognized by any quality control mechanism and that the mutant is transported to the membrane in a preferred manner.

## Supplementary Information

**Additional file 1.** List of genes included in the TruSight Cardio Panel (Illumina).

## Data Availability

The raw data are available upon request from the corresponding author. The genetic variant detected during this study is available in the NCBI dbSNP database, rs1331765859. The human reference genome GRCh37/hg19 was used as reference data set in this study.

## Abbreviations

AF: Atrial fibrillation
BrS: Brugada syndrome
DCM: Dilated Cardiomyopathy
ECG: Electrocardiogram
EGFP: Enhanced green fluorescent protein
HEK293: Human embryonic kidney cells (cell line 293)
LQTS: Long QT syndrome
LV-EF: Left ventricular ejection fraction
MRI: Magnetic resonance imaging
MT: Mutant
NGS: Next-generation sequencing
SCD: Sudden cardiac death
SSS: Sick sinus syndrome
WT: Wild type
